# Predictors of Noncompliance to Antihypertensive Therapy among Hypertensive Patients Ghana: Application of Health Belief Model

**DOI:** 10.1155/2018/4701097

**Published:** 2018-06-19

**Authors:** Yaa Obirikorang, Christian Obirikorang, Emmanuel Acheampong, Enoch Odame Anto, Daniel Gyamfi, Selorm Philip Segbefia, Michael Opoku Boateng, Dari Pascal Dapilla, Peter Kojo Brenya, Bright Amankwaa, Evans Asamoah Adu, Emmanuel Nsenbah Batu, Adjei Gyimah Akwasi, Beatrice Amoah

**Affiliations:** ^1^Department of Nursing, Faculty of Health and Allied Sciences, Garden City University College (GCUC), Kenyasi, Kumasi, Ghana; ^2^Department of Molecular Medicine, School of Medical Science, Kwame Nkrumah University of Science and Technology (KNUST), Kumasi, Ghana; ^3^School of Medical and Health Science, Edith Cowan University, Joondalup, Australia; ^4^Department of Medical Laboratory Technology, Faculty of Allied Health Sciences, KNUST, Ghana; ^5^Department of Nursing, Kintampo Municipal Hospital, Kintampo, Ghana; ^6^Department of Community Health, School of Medical Sciences, KNUST, Ghana

## Abstract

This study determined noncompliance to antihypertensive therapy (AHT) and its associated factors in a Ghanaian population by using the health belief model (HBM). This descriptive cross-sectional study conducted at Kintampo Municipality in Ghana recruited a total of 678 hypertensive patients. The questionnaire constituted information regarding sociodemographics, a five-Likert type HBM questionnaire, and lifestyle-related factors. The rate of noncompliance to AHT in this study was 58.6%. The mean age (SD) of the participants was 43.5 (±5.2) years and median duration of hypertension was 2 years. Overall, the five HBM constructs explained 31.7% of the variance in noncompliance to AHT with a prediction accuracy of 77.5%, after adjusting for age, gender, and duration of condition. Higher levels of perceived benefits of using medicine [aOR=0.55(0.36-0.82),p=0.0001] and cue to actions [aOR=0.59(0.38-0.90),p=0.0008] were significantly associated with reduced noncompliance while perceived susceptibility [aOR=3.05(2.20-6.25), p<0.0001], perceived barrier [aOR=2.14(1.56-2.92), p<0.0001], and perceived severity [aOR=4.20(2.93-6.00),p<0.0001] were significantly associated with increased noncompliance to AHT. Participant who had completed tertiary education [aOR=0.27(0.17-0.43), p<0.0001] and had regular source of income [aOR=0.52(0.38-0.71), p<0.0001] were less likely to be noncompliant. However, being a government employee [aOR=4.16(1.93-8.96), p=0.0002)] was significantly associated increased noncompliance to AHT. Noncompliance to AHT was considerably high and HBM is generally reliable in assessing treatment noncompliance in the Ghanaian hypertensive patients. The significant predictors of noncompliance to AHT were higher level of perceived barriers, susceptibility, and severity. Intervention programmes could be guided by the association of risk factors, HBM constructs with noncompliance to AHT in clinical practice.

## 1. Background

Hypertension (HTN) is a major risk factor for cardiovascular diseases such as heart failure, myocardial infarction, and stroke [1–4]. Cardiovascular diseases are the major cause of mortality among adults globally [1] of which about 50% can be ascribed to complications of HTN [5]. Developing countries account for almost 80% of these deaths [5]. Among the list of noncommunicable diseases plaguing the general Ghanaian population, HTN is said to be the most prevalent [6, 7]. Efforts geared towards improving lifestyles, controlling lifestyle-related major cardiovascular risk factors, absolutely will contribute to the prevention of cardiovascular diseases [1, 5]. The Ghana Health Service (GHS) also estimated that the prevalence rate of HTN in Ghana among adults of 18 years and above was 29.9 per cent for males and 27.6 per cent for females (GHS, 2014). It has been reported that blood pressure (BP) control among hypertensives in Ghana is largely poor due to noncompliance to therapy [8]. Noncompliance to antihypertensive therapy (AHT) involves the interplay of the healthcare-provider/health system, therapy, condition, client, and socioeconomic factors [3, 8, 9].

The annual health report of the Kintampo Municipality continues to show HTN ranking among the top 10 diseases over the past 5 years (Municipal Health Year Report, 2015). Moreover, it was the fourth leading cause of death in the municipality. Failure to maintain a well-controlled blood pressure (BP) has been mainly attributed to noncompliance to AHT [2, 3]. To the best of our knowledge, no published study has been conducted to determine noncompliance among hypertensives in the municipality. The health belief model (HBM) is one of the most classical theories devised by social psychologists to describe social behaviour as well as public participation in medical programs [10]. The model was further extended to the study of range of health behaviours which included dietary behaviour, smoking, contraceptives, physical activities alcohol use and drinking, and obstetrics outcome [11–13]. The model contains several cognitive constructs that predict why people take actions to control their illness including perceived susceptibility, perceived severity, perceived benefits, perceived barriers, and cues to actions. HBM has strength to allow patient diagnosed with HTN to consider the benefits to be gained from compliance (behaviour), is worth the cost, and also assess his or her severity and vulnerability to HTN complications before making the decision [14]. There is limited research evidence on the implications of health behaviour from low income countries such as Ghana, although the applications of this model have been widely used in developed countries. Several studies have investigated the prevalence of antihypertensive compliance and its determinants in Ghana [15–18]; no study has focused on the applicability of HBM. It was against this background that the study sought to evaluate noncompliance to AHT among Ghanaian hypertensive patients using HBM.

## 2. Material and Methods

### 2.1. Study Design and Setting

This study was a cross-sectional descriptive study among hypertensives attending the Hypertension Clinic at the Kintampo Municipal Hospital. The Kintampo Municipal Hospital is the major source of health care for the inhabitants of the Kintampo Municipality. The Hypertension Clinic was established in April 2015 under the auspices of the Kintampo Municipal Health Directorate and operates only on Wednesdays. The clinic is being run by a medical doctor, a physician assistant, a general nurse, an enrolled nurse, and two health extension workers.

### 2.2. Study Population and Subject Selection

The targeted population was hypertensive patients who were on antihypertensive therapy and attending the Hypertension Clinic. Simple random sampling technique was used to recruit six hundred and seventy-eight (678) hypertensive patients who consented for the study at the Hypertension Clinic at the Kintampo Municipal Hospital. Each hypertensive patient was given a number and then a table of random numbers to decide which patient to include. Depending on the total number of patients per each clinic, a range of 20 to 24 patients were randomly sampled weekly until sample size was achieved. Hypertensive patients who had been diagnosed or were on medication for hypertension for one year or more were included in the study as well as hypertensive patients aged 30 years and above. Moreover, hypertensives with or without other existing comorbidities such as diabetes, cardiovascular diseases, and renal diseases were also included in the study. Hypertensive patients who did not consent and were seriously ill (too sick to be interviewed) were excluded from the study

### 2.3. Data Collection Tool

For validity and reliability of the study, a pilot study was conducted using the research instrument. This was aimed at testing the strength of the research instrument to elicit the needed responses for the study. The pilot study was carefully evaluated by the researchers and an expert in the field of research. Necessary amendments were made and the resulting questionnaire was used for the main data collection process. Reliability coefficients ranging from 0.00 to 1.00, with higher coefficients indicating higher levels of reliability, were used to determine the validity and the reliability of the questionnaire. The reliability coefficients for all the questions were 0.903.

The questionnaire developed for this study was adopted from studies conducted by Joho [19]. It was made up of three sections. Section A collects the sociodemographic characteristics data of the participants. Section B was designed to collect information on treatment compliance, which comprised both medication regimen compliance and lifestyle modification. Medication regimen compliance was composed of 8 items, asking how often they forgot to take their medicine, did they stop taking their medicine because they felt better, because they felt worse, because they believed that medicine was ineffective, because they feared side effects, because they tried to avoid addiction, because of religious beliefs or they were using traditional medicine, and because of cost of medication. The responses were measured on a 4-point Likert scale (every day, frequently, rarely, or never). Lifestyle compliance composed of 5 items which included how often they did smoke, consumed alcohol, engaged in physical exercise, ate table salt, and ate meat with high animal fats. Participants were asked to respond to the single question based on a 4-point Likert scale: how often do desirable or undesirable behaviours related to control of hypertension. The responses were every day, frequently, rarely, or never. The responses were (1) every day, (2) frequently, (3) rarely, or (4) never. Some questions were set such that the highest score did not reflect the worst scenario of noncompliance. To resolve this, scores were reversed. For instance, how often do you engage in physical exercise: (4) every day, (3) frequently, (2) rarely, or (1) never. Section C constituted the HBM variables which include perceived severity of hypertension measured by six items which included whether BP was a serious problem, worried about their HTN condition, getting HTN was serious, getting HTN complications was very dangerous, and dying due HTN complication was dangerous; perceived susceptibility of being at risk of hypertension complications measured by six items, thus having stroke, developing visual impairment, heart problems, kidney problems, becoming burden for family, and career being negatively affected; perceived benefit treatments were each measured by six items which were keeping their BP under control, increasing their quality of life, increasing their sense of well-being, protecting them from complications, avoiding added financial burden to treat complications, and decreasing my chance of dying; and cues to action were measured by seven items. Participants were then asked to respond strongly agree (4), agree (3), disagree, (2) or strongly disagree (1). The remainder which is perception of barriers was also measured by five items, ineffective of the medicine to stabilize their BP, lack of motivation because they cannot be cured, not having enough time to exercise, lack of discipline to comply with dietary restriction, and lack of motivation to stop smoking. The responses were ‘not at all' (1), ‘to some extent' (2), ‘to a larger extent' (3), or ‘very much extent' (4). The 13 items measuring treatment compliance and life style compliance were added up to get sum index with a distribution ranging from 23 to 52 with mean 44.30 (SD =5.55), and the median split was used (46.0), which was dichotomized into two groups, i.e., 1 = those who are nontreatment compliant and 0 = treatment compliant which was 23-45 and 46-52. The variables comprising the number of items measuring compliance in the HBM were added up to get sum index with a distribution range, and the median split was used as a cut point. Dichotomization was done into two frequency groups, those who had low perceived severity, susceptibility, benefits, and vice versa. The entire questionnaire was available in English version but interviewed carefully with the proper translation of the official local language of the study population. The responses of the participants were translated back to English in the correct meaning as was interpreted.

### 2.4. Ethical Consideration

Approval for this study was obtained from Human Research, Publication and Ethics of the School of Medical Sciences (SMS), Kwame Nkrumah University of Science and Technology (KNUST) (CHRPE/AP/213/16). Participation was voluntary and written informed consent was obtained from each participant. Hypertensive clients were also given a short exposition on the essence of the study and their role to play to make it a success. The clients who were prospective subjects for the study were made to understand that the research was solely for academic purposes, and that no information would be handled with anything short of full privacy and confidentiality.

### 2.5. Statistical Analysis

Data was entered into excel worksheet and analysed using the statistical package for social sciences version (SPSS) 23.0. Continuous variables were expressed as mean ± SD for normal distributed data and median (interquartile range) for not normally distributed data, respectively. Frequency distributions were done as sociodemographic data and then bivariate analysis using chi-squire for association of sociodemographic characteristics and HBM with treatment noncompliance; and Pearson correlation between HBM variables was done. Logistic regressions were done with treatment noncompliance as the outcome variable and the rest of HBM variables as predictors. Statistical significance was assumed at p<0.05.

## 3. Results

The mean age (SD) of the participants was 43.5(±5.2) years and median duration of the disease was 2 years. Majority of them were males (50.7%). Considerable proportions of the participants had no education (34.2%), were married (66.7%), and were private employees (50.8%). Most of the participants have had the condition for less than 5 years (72.0%). Moreover, more than half of the participants (60.8%) did not have regular income** [Table 1].**

**Figure 1:**
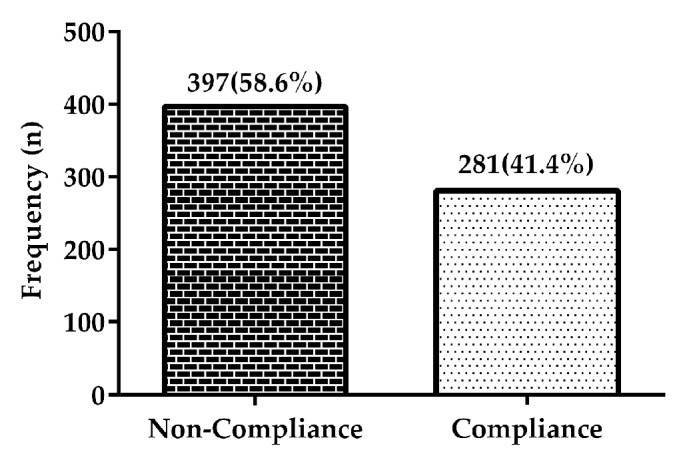
Frequency distribution of noncompliance and compliance among participants.

Three hundred and ninety-seven (58.6%) participants were not compliant to AHT, while 41.4% complied** [Figure 1].**

Participants with high cues to actions and perceived benefits had reduced odds for noncompliance [aOR=0.59(0.38-0.90), p=0.0008); OR=0.55(0.36-0.82), p=0.0001)]. Moreover, participants with high perceptions of severity had significantly increased odds for noncompliance [aOR=4.20(2.93-6.00), p<0.0001)]. Participant with high perception of susceptibility [aOR=3.05, (2.20-4.25), p<0.0001)] and high perceived barriers [aOR=2.14(1.56-2.92), p<0.0001)] were more likely to be noncompliant to AHT** [Table 2].**

As shown in**Table 3**, treatment noncompliance showed significant and positive association with perceived severity (r=0.19, p<0.0001) and susceptibility (r=0.33, p<0.001). Furthermore, treatment noncompliance was significantly negatively associated benefits (r= -0.449, p<0.0001).

Participant who had completed tertiary education had significantly reduced odds for noncompliance to AHT [aOR=0.27(0.17-0.43), p<0.0001)]. However, being a government employee [aOR=4.16(1.93-8.96), p=0.0002)] was significantly associated with increased likelihood of being noncompliant to AHT. Participants who had regular source of income had lower odds for noncompliance to AHT [aOR=0.52(0.38-0.71), p<0.0001)]. Being male [aOR=1.33(0.98-1.81), p=0.074)], being divorced [aOR=2.00(0.98-4.09, p=0.077)], and duration of HTN 5-10 years [OR=1.37(0.96-1.95), p=0.092) had significant association with noncompliance to AHT** [Table 4]**.

Lifestyle-related factors such as number of medicine taken (p=0.002), history of smoking (p<0.0001), and history of alcohol consumption (p<0.0001) had statistically significant association with noncompliance to AHT. Moreover, considerable proportions of the participants who had health compliant other than HTN (73.0%) and those on two different drugs (56.9%) were noncompliant to AHT** [Table 5]**.

Multiple logistic regression analysis showed significant model fit for the data (F=24.7, p< 0.0001). The amount of variance in noncompliance to AHT which is explained by the predictors is 31.7 % (R^2^=0.317) with perceived barrier being the strongest predictor of noncompliance to AHT (*β* = 0.780, p< 0.0001). Positive beta coefficient indicates a positive association between perceived barriers and noncompliance to AHT** [Table 6]**.

## 4. Discussion

This study determined noncompliance to AHT and its associated factors among hypertensive patients in the Kintampo Municipality using HBM questionnaire. The rate of noncompliance to AHT was 58.6% which indicates that more than half of the participants were not complaint to their medication. This observed prevalence rate is lower compared to other cross-sectional studies done in the Ghanaian setting by Kretchy et al. [15] and Buabeng et al. [16] but higher compared to a study by Jambedu among Ghanaian hypertensive patients [17]. The possible explanations for these disparities in findings could be due to the differences in sample size and the type of questionnaire used, as previous studies did not employ the HBM. Conversely, the observed noncompliance rate in this current study is comparable to range of reports from previous cross-sectional studies done elsewhere in Africa [8, 20–22]. These results highlight the fact that noncompliance to AHT is very common in the Ghanaian setting. The high noncompliance rate reported in this study is also consistent with some cross-sectional studies in Pakistan [23, 24], China [25, 26], and Iran [27].

Several retrospective and prospective studies have provided extensive support for the HBM in evaluating a range of health-related behaviours, although some other studies do not fully support this theory [11, 28, 29]. The HBM performed fairly well in predicting noncompliance to AHT among Ghanaian hypertensive patients in this study. The amount of variance in treatment noncompliance which is explained by the five constructs was 31.7 % with an overall prediction accuracy of 77.5% after adjusting age, gender, and duration of condition. This indicates that HBM is generally reliable in predicting noncompliance to AHT and framing intervention measures to reduce noncompliance to AHT among Ghanaian hypertensive patients.

Perceived barrier was observed to be the strongest predictor of treatment noncompliance which is consistent with finding observed by Day et al. [30]. It was also observed that higher levels of perceived barriers were associated with increased odds of noncompliance to AHT. This result corresponds closely to finding from previous study conducted by Haynes et al., who reported that perceived barrier was the strongest predictor of noncompliance to AHT [31]. Higher perceived benefit of using medicine and cue to actions significantly correlated with reduced odds of noncompliance to AHT. These findings concur with current literature on hypertensive medication adherence among Chinese [25, 32]. Thus, intervention measures suitable for Ghanaian hypertensive patients should mainly focus on reducing perceived susceptibility, perceived severity, and perceived barriers but increase cues to action and perceived benefits.

In this study, the average age of all included hypertensive patients was 43.5 years indicating high prevalence of hypertension in younger adults compared to the elderly. This is inconsistent with studies by Almas et al. [33] and Okwuonu et al. [20], where mean age within the fifties were reported, depicting higher prevalence of hypertension among the elderly. However, current literature indicates that the prevalence rate of hypertension in the Ghanaians population is higher in the 40-55 years age groups [16, 34, 35]. Our current study confirms this change of trend, where now the prevalence is higher in those in their forties and fifties. The high prevalence in the young adults in our population could be due to change in lifestyle and adaptation of Westernized diets, full of salts and fats that could predispose them to hypertension. Moreover, logistic regression models in this study showed that participants within 41-50 years had increased odds for noncompliance to AHT. The majority of the previous studies had shown that age is related to compliance, although a few researchers found age not to be a factor causing noncompliance [36–38]. Patients in this age ranges (middle-aged patients) always have other priorities in their daily life; due to their work and other commitments, they may not be able to attend for treatment or spend a long time waiting for clinic appointments

Participants who have had tertiary education had significant reduced odds for noncompliance to AHT in this study. This is consistent with studies by Okuno et al. [39], Ghods and Nasrollahzadeh [40], and Yaruz et al. [41] who found that patients with higher educational level have higher compliance to their medication. Intuitively, it may be expected that patients with higher educational level should have better knowledge about the disease and therapy and therefore be more compliant. Sufficient knowledge about hypertension in patients has been associated with greater medication adherence and better BP control in previous studies [33, 42]. Other studies have also reported contrasting results where high level of education was associated with low compliance [43, 44]

Findings from this study showed that government and private employees had significant increased odds for noncompliance to AHT. It is assumed that patients who are employed can afford the cost of medication and treatment and hence more likely to be compliant to medication. However, high noncompliance was observed among employed participants and this could be attributed to the busy work schedules and patients having less time for self-care or management [45, 46]. Other studies have also reported contrasting results whereby prevalence of noncompliance to AHT was found to be higher among unemployed individuals with low socioeconomic status [24]. Furthermore, participants with regular source of income had lower odds for noncompliance to AHT. This result is supported by cross-sectional studies conducted in Egypt, by Awad et al. [47] and in Saudi Arabia and Sudan by EL-Zubier [48] who reported that the insufficient income will possibly affect compliance principally if patient is receiving numerous drugs or the drug is expensive. Distribution of participants by reasons of compliant to antihypertensive medication was determined. The main reason for noncompliance reported by participants was cost of medication, stopping medication when feeling well, fear of side effects, forgetfulness, and use of traditional medicine. These findings are supported by reports from previous cross-sectional studies conducted by Almas et al. [33] and Hashim et al. [49].

Although findings in this study concur with reports from other studies and highlight the burden of noncompliance to AHT in the Kintampo municipality and probable associated factors such as health beliefs and lifestyle, there were some limitations. This was a cross-sectional study conducted with small sample size which limited our ability to explain the causal correlations between variables and noncompliance. All participants came from one municipality, and thus, the findings may not represent all of Ghana. The current HBM only includes five cognitively based constructs. It does not consider the emotional, environmental, and social components of health behaviour, which should be added to the HBM in future studies.

## 5. Conclusion

The study showed that noncompliance to AHT was considerably high and HBM is generally reliable in assessing treatment noncompliance in the Ghanaian hypertensive patients. The study further identified sociodemographic characteristics such as educational level, occupational status, and regular source of income to be significantly associated with noncompliance in the hypertensive patients higher level of perceived barriers; susceptibility and severity were significant predictors of noncompliance: therefore, intervention programmes for noncompliance can be directed towards a greater involvement of increased perceived benefits, cues to action, and personality characteristics such as locus of control.

## Figures and Tables

**Table 1:** Sociodemographic characteristics of study participants.

**Variables**	**Frequency (n=678)**	**Percentages (%)**
Age (years) (Mean ± SD)	43.5±6.2	

**Age Groups**		
31-40	301	44.4%
41-50	264	38.9%
51-60	113	16.7%
**Gender**		
Male	344	50.7%
Female	334	49.3%
**Marital Status**		
Single	90	13.3%
Married	452	66.7%
Divorced	51	7.5%
Separate	38	5.6%
Widowed	30	4.4%
Cohabiting	17	2.5%
**Education Level**		
Uneducated	232	34.2%
Basic	203	30.0%
SHS	115	16.9%
Tertiary	128	18.9%
**Occupational Status**		
Government employee	230	33.9%
Private employee	344	50.7%
Self-employed	65	9.6%
Student	5	0.7%
Unemployed	34	5.0%
**Duration of Condition(years)**		
<5	488	72.0%
5-10	179	26.4%
>10	12	1.6%
**Regular Source Income**		
No	412	60.8%
Yes	266	39.2%
Duration of Condition (years) (Median, IQR)	2.0(1.0-5.0)	
Duration of Treatment (years) (Median, IQR)	2.0(1.0-5.0)	

***SD: standard deviation, IQR: interquartile range, JHS: junior high school, and SHS: senior high school.***

**Table 2:** Association of constructs of HBM with participant's treatment compliance.

**Variables **	**Non-compliance**	**Compliance**	**p-value**	**aOR (95%CI)**
**Cues to Action **			**0.0008**	
Low	226(56.9%)	123(43.8%)		1
High	171(43.1%)	158(56.2%)		0.59(0.38-0.90)
**Perception of benefits**			**0.0001**	
Low	233(58.7%)	123(43.8%)		1
High	164(41.3%)	158(56.2%)		0.55(0.36-0.82)
**Perception of severity**			**<0.0001**	
Low	201(50.6%)	228(81.1%)		1
High	196(49.4%)	53(18.9%)		4.20(2.93-6.00)
**Perception of susceptibility**			**<0.0001**	
Low	188(47.4%)	206(73.3%)		1
High	209(52.6%)	75(26.7%)		3.05(2.20-4.25)
**Perception to barriers **			**<0.0001**	
Low	179(45.1 %)	179(63.7%)		1
High	218(54.9%)	102(36.3%)		2.14(1.56-2.92)

***aOR: adjusted odds ratio; CI: confidence interval; p<0.05 is statistically significant; and 1*** *∗* ***reference category.***

**Table 3:** Partial correlation between HBM constructs controlling for age, gender, and duration of disease.

Variables		1	2	3	4	5	6
1.Treatmen non-compliance	r	-	0.19	0.33	-0.21	-0.449	-0.012
	p-value		**<0.0001**	**<0.0001**	**<0.0001**	**<0.0001**	0.820
2.Perceived Severity	r		-	0.539	-0.013	-0.294	0.087
	p-value			**<0.0001**	0.808	**<0.0001**	0.099
3.Perceived Susceptibility	r			-	0.067	-0.538	0.339
	p-value				0.206	**<0.0001**	**<0.0001**
4.Perceived Benefits	r				-	0.018	0.464
	p-value					0.735	**<0.0001**
5.Perceived Barriers	r					-	0.111
	p-value						**0.036**
6.Cues to Action	r						-
	p-value						

***r: correlation coefficient; p<0.05 is statistically significant.***

**Table 4:** Sociodemographics of study participants and relation to compliance.

**Variables **	**Non-compliance**	**Compliance**	**X** ^**2**^ **, df**	**P-value**	**aOR (95% CI)**	**p-value**
**Age Groups (years)**			1.3,2	0.519		
31-40	167(42.1%)	134(47.7%)			1	
41-50	164(41.3%)	100(35.6%)			1.32(0.94-1.84)	0.124
51-60	66(16.6%)	47(16.7%)			1.13(0.73-1.75)	0.657
**Gender**						
Male	214(53.9%)	130(46.2%)			1.33(0.98-1.81)	0.074
Female	187(46.1%)	151(53.8%)			1	
**Marital Status**			3.6, 6	0.724		
Single	45(11.3%)	45(16.0%)			1	
Married	264(66.5%)	189(67.3%)			1.40(0.88-2.20)	0.163
Divorced	34(8.6%)	17(6.0%)			2.00(0.98-4.09)	0.077
Separate	23(5.8%)	15(5.4%)			1.53(0.71-3.31)	0.334
Widowed	20(5.0%)	9(3.2%)			2.22(0.91-5.41)	0.089
Cohabiting	11(2.8%)	6(2.1%)			1.83(0.62-5.39)	0.301
**Educational Level**			20.8, 4	**<0.0001**		
Unschooled	154(38.9%)	77(27.4%)			1	
Basic	132(33.2%)	72(25.6%)			0.92(0.61-1.36)	0.686
SHS	66(16.6%)	49(17.4%)			0.67(0.42-1.07)	0.098
Tertiary	45(11.3%)	83(29.6%)			0.27(0.17-0.43)	**<0.0001**
**Occupational Status**			13.1, 4	**0.011**		
Government employee	153(38.5%)	77(27.4%)			4.16(1.93-8.96)	**0.0002**
Private employee	201(50.6%)	143(50.9%)			2.94(1.39-6.22)	**0.006**
Self-employed	32(8.1%)	32(11.4%)			2.09(0.88-4.99)	0.134
Student	0(0.0%)	6(2.0%)			-	-
Unemployed	11(2.8%)	23(8.2%)			1	
**Duration of Condition(years**)			1.8,2	0.411		
<5	277(69.8%)	211(75.1%)			1	
5 -10	115(29.0%)	64(22.8%)			1.37(0.96-1.95)	0.092
>10	5(1.2%)	6(2.1%)			0.63(0.19-2.11)	0.544
**Regular Source Income**			10.0,1	**0.007**		
No	267(67.2%)	145(51.6%)			1	
Yes	130(32.8%)	136(48.4%)			0.52(0.38-0.71)	**<0.0001**

***X*** ^***2***^ **:* Chi-square, df: degree of freedom, aOR: adjusted odds ratio, CI: confidence interval, JHS: junior high school, and SHS: senior high school; p<0.05 is statistically significant;*** **∗** ***1 reference category.***

**Table 5:** Association of lifestyle-related factors with treatment noncompliance.

**Variables **	**Non-compliance**	**Compliance**	**P-value**
Health complaint other than HTN			0.069
Yes	290(73.0%)	223(79.3%)	
No	107(27.0%)	58(20.7%)	
Number of Medicine taken			**0.002**
1	38(9.6%)	8(2.9%)	
2	226(56.9%)	177(63.0%)	
3	115(29.0%)	90(32.0%)	
≥4	18(4.5%)	6(2.1%)	
History of smoking			**<0.0001**
No	344(86.6%)	279(99.3%)	
Yes	53(13.4%)	2(0.7%)	
History of alcohol consumption			**<0.0001**
No	269(67.8%)	257(91.4%)	
Yes	128(32.2%)	24(8.6%)	

***P<0.05 is statistically significant.***

**Table 6:** Cross-sectional association and predictability of HBM variables for noncompliance.

**Health belief model variables**	**Beta**	**SE**	**aOR (95%)**	**P-value**
Perceived Severity	-0.007	0.078	0.99(0.25-1.78)	0.933
Perceived susceptibility	0.142	0.067	1.15(0.75-2.72	**0.034**
Perceived benefits	-0.414	0.099	0.66(0.09-1.62)	**<0.0001**
Perceived barriers	0.780	0.115	2.18(1.09-4.12)	**<0.0001**
Cues to actions	-0.006	0.062	0.98(0.22-1.64)	0.925

***R*** ^***2***^ ***=0.317, F=24.7(p<0.0001), SE: standard error, p<0.05 is statistically significant, and aOR: adjusted odd ratio.***

## Data Availability

All relevant data are within the article.
